# The value of a new prognostic model developed by lymphocyte-monocyte ratio and platelet-monocyte ratio in peripheral T-cell lymphoma

**DOI:** 10.1186/s12935-021-02275-2

**Published:** 2021-10-29

**Authors:** Yan Zhang, Yuanfei Shi, Huafei Shen, Lihong Shou, Qiu Fang, Xiaolong Zheng, Mingyu Zhu, Xin Huang, Jiansong Huang, Li Li, De Zhou, Lixia Zhu, Jingjing Zhu, Xiujin Ye, Jie Jin, Wanzhuo Xie

**Affiliations:** 1 Department of Hematology, College of Medicine, Affiliated Huzhou Hospital, Zhejiang University, No. 1558 North Third Ring Road, Huzhou, 313000 Zhejiang China; 2 Department of Hematology, College of Medicine, the First Affiliated Hospital, Zhejiang University, No. 79 Qingchun Road, Hangzhou, 310003 Zhejiang China; 3 Key Laboratory of Hematologic Malignancies, Diagnosis and Treatment, Hangzhou, 310003 Zhejiang China

**Keywords:** Peripheral T-cell lymphoma, Lymphocyte, Platelet, Monocyte, Prognosis, Tumor micro-environment, Immunity

## Abstract

Peripheral T-cell lymphoma(PTCL) is a group of lymphoproliferative tumors originated from post-thymic T cells or mature natural killer (NK) cells. It shows highly aggressive clinical behaviour, resistance to conventional chemotherapy, and a poor prognosis. Although a few prognostic models of PTCL have been established in retrospective studies, some high-risk patients still can not be screened out. Therefor we retrospectively studied 347 newly diagnosed PTCL patients and assessed the prognostic role of lymphocyte-monocyte ratio (LMR) and platelet-monocyte ratio (PMR) in the complete response (CR) and survival of PTCL patients. Patients with LMR ≤ 1.68 and PMR ≤ 300 achieved a lower CR rate and a poor survival. In multivariate analysis, LMR ≤ 1.68 (HR = 1.751, 95% CI 1.158–2.647, p < 0.05) and PMR ≤ 300 (HR = 1.762, 95% CI 1.201–2.586, p < 0.05) were independently associated with short survival. On this basis, a new prognostic model of PTCL was established to screen out high-risk patients. In our "Peripheral Blood Score (PBS)" model, three groups were identified at low risk (178 patients, 51.3%, score 0), intermediate risk (85 patients, 24.5%, score 1), and high risk (84 patients, 24.2%, score 2), having a 1-year OS of 86%, 55.3% and 22.6% (p < 0.05), and a 3-year OS of 43.4%, 20% and 13.1% (p < 0.05), respectively. Optimal strategies for identifying high-risk patients with PTCL are urgently needed. Our new PBS model is simple, inexpensive and widely available to screen out the high risk patients.

## Introduction

Peripheral T-cell lymphoma (PTCL) is a group of rare hematological malignancies with heterogeneous morphological and biological characteristics. The overall manifestations are high invasive, short survival and poor prognosis. The total incidence rate is 0.5–2, per 100,000 persons per year, and account for about 10% of all non-Hodgkin’s lymphomas (NHL) in Western countries [1]. However, the incidence of PTCL in Asia is higher, accounting for 25–30% of NHL in China [2]. Nowadays, the internationally recommended first line therapy is still anthracycline-based chemotherapy. But the complete response (CR) rate after chemotherapy is only 40–60%, with overall survival (OS) of 30–40%. Most patients face the problem of short-term recurrence and the median OS of these patients without stem cell transplantation were only 5.5 months [3–5]. Once the disease relapses or progresses, patients will lose effective treatment measures.

According to the WHO classification, PTCL can be further divided into many pathological subtypes. The most common subtypes include PTCL not otherwise specified (PTCL-NOS), extra-nodal natural killer (NK)/T cell lymphoma, nasal type (ENKTL), angioimmunoblastic T-cell lymphoma (AITL), anaplastic lymphoma kinase positive anaplastic large cell lymphoma (ALK + ALCL) and anaplastic lymphoma kinase negative anaplastic large cell lymphoma (ALK− ALCL) [6]. In addition, there are some rare subtypes, such as monomorphic epitheliotropic intestinal T-cell lymphoma (MEITL), subcutaneous panniculitis like T-cell lymphoma (SPTCL), mycosisfungoides/Sezary’s syndrome (MF/SS), Hepatosplenic T-cell lymphoma (HSTCL) and so on. Due to its rarity and heterogeneity, the prognosis of PTCL were less studied.

Over the past few decades, a number of prognostic models based mainly on clinical variables have been put forward, among which the International Prognostic Index (IPI) scoring model based on the data of patients with diffuse large B-cell lymphoma was earlier and more widely used on PTCL patients[3]. The Intergruppo Italiano Linfomi (now Fondazione Italiana linfomi, FIL) performed a large study on 385 patients diagnosed and treated in the 1990s and defined the Prognostic Index for PTCL-unspecified (PIT), in which age, ECOG, LDH level and bone-marrow involvement were independent predictors of OS in PTCL-NOS patients [7]. The PIT divided the patients into four different risk groups: low-risk (no adverse factors), intermediate (1 adverse factor), intermediate-high (2 adverse factors) and high (3–4 adverse factors) [7]. The 5-year OS were 62.3%, 52.9%, 32.9% and 18.3%, respectively (P < 0.05). The PIT can stratify patients more effectively than IPI [7]. A common limitation of the above models is the complexity of their use.

Systemic inflammatory response and host immunity played an important role in promoting the clinical courses of tumors and determining the prognosis of tumor patients [8]. According to the previous studies, LMR was closely related to the prognosis of esophageal squamous cell carcinoma, malignant melanoma, bladder cancer and ovarian clear cell cancer [9–12]. Similarly, platelet count and PMR were closely related to the occurrence of acute exacerbation of chronic obstructive pulmonary disease, pulmonary embolism and prognosis of various tumors [13]. In hematological malignancies, LMR and PMR are also bound up with poor prognosis of diffuse large B-cell lymphoma (DLBCL) and Follicular lymphoma (FL) [14–16]. However, there are few reports about the response and prognosis of LMR and PMR in PTCL patients.

Here, we retrospectively analyzed 347 patients with primary PTCL in a single center and found that patients with PMR ≤ 300 and LMR ≤ 1.68 were closely related to poor response and low survival rate. On this basis, established a peripheral blood score (PBS) model for identification of high-risk PTCL patients. The patients were divided into low-risk, intermediate-risk and high-risk groups. 44 patients who met the enrollment Requirements in another center were substituted into the PBS model for verification. PBS model can distinguish some patients with poor prognosis.

## Materials and methods

### Patients and characteristics

This is a single center retrospective study. A total of 347 patients with PTCL newly diagnosed in the First Affiliated Hospital of Zhejiang University School of Medicine from January 2011 to October 2019 were included. The final observation time was January 2020, and the median follow-up time was 18 months (rang: 0–108 months). The inclusion criteria were as follows: (1) Age ≥ 15 years; (2) The pathological diagnosis was consistent with PTCL; (3) Newly diagnosed and no chemotherapy before clinical data were collected; (4) Complete clinical data; (5) At least two cycles of treatment were given. Although the treatment plan were not completely unified, all the patients in our study received cyclophosphamide-doxorubicin-vincristine-prednisone (CHOP) or CHOP-like chemotherapy regimen, and all ENKTL patients were treated with chemotherapy combined with Pegaspargase. All procedures involving human participants in our study were conducted in accordance with the Helsinki declaration.

We collected the medical records, physical examinations, laboratory results, pathological reports and radiological results of these patients through electronic medical records, and re analyzed the clinical data of them. All baseline data are presented in Table 1. Follow-up was performed by making phone calls. The absolute counts of lymphocytes, monocytes and platelets were obtained from a standard complete blood count (CBC) performed at diagnosis. LMR was the absolute count of lymphocyte divided by the absolute count of monocyte. PMR was the ratio of platelet absolute count to monocyte absolute count. OS was defined as the time from diagnosis to death for any reasons or last follow-up.

**Table 1:** Characteristics of 347 patients with PTCL based on the LMR and PMR

Characteristic	Total	LMR ≤ 1.68	LMR > 1.68	p value	PMR ≤ 300	PMR > 300	p value
	(n = 347)	(n = 127)	(n = 220)		(n = 126)	(n = 221)	
Age				0.063			0.202
< 60 years	222(64.0)	73(57.5)	149(67.7)		75(59.5)	147(66.5)	
≥ 60 years	125(36.0)	54(42.5)	71(32.3)		51(40.5)	74(33.5)	
Sex				0.482			0.289
Male	229(66.0)	87(68.5)	142(64.5)		88(69.8)	141(63.8)	
Female	118(34.0)	40(31.5)	78(35.5)		38(30.2)	80(36.2)	
IPI				< 0.05^*^			< 0.05^*^
0–2	154(44.4)	29(22.8)	125(56.8)		27(21.4)	127(57.5)	
3–5	193(55.6)	98(77.2)	95(43.2)		99(78.6)	94(42.5)	
ECOG				< 0.05^*^			< 0.05^*^
0–2	241(69.5)	66(52.0)	175(79.5)		64(50.8)	44(19.9)	
3–5	106(30.5)	61(48.0)	45(20.5)		62(49.2)	177(80.1)	
Stage				< 0.05^*^			< 0.05^*^
I–II	65(18.7)	1(0.8)	64(29.1)		6(4.8)	59(26.7)	
III–IV	282(81.3)	126(99.2)	156(70.9)		120(95.2)	162(73.3)	
B symptoms				< 0.05^*^			< 0.05^*^
Yes	203(58.5)	97(76.4)	106(48.2)		94(74.6)	109(49.3)	
No	144(41.5)	30(23.6)	114(51.8)		32(25.4)	112(50.7)	
Histological subtype				0.330			0.018^*^
PTCL,NOS	111(32.0)	45(35.4)	66(30.0)		51(40.5)	60(27.1)	
ENKTL	113(32.5)	32(25.2)	81(36.8)		31(24.6)	82(37.1)	
AITL	70(20.2)	27(21.2)	43(19.5)		29(23.0)	41(18.6)	
ALCL,ALK +	20(5.8)	10(7.9)	10(4.5)		8(6.3)	12(5.4)	
ALCL,ALK-	21(6.0)	7(5.5)	14(6.3)		3(2.4)	18(8.1)	
MEITL	4(1.2)	3(2.4)	1(0.5)		1(0.8)	3(1.4)	
SPTCL	5(1.4)	2(1.6)	3(1.4)		1(0.8)	4(1.8)	
HSTCL	2(0.6)	1(0.8)	1(0.5)		2(1.6)	0(0.0)	
MF/SS	1(0.3)	0(0.0)	1(0.5)		0(0.0)	1(0.5)	
Bone marrow Involvement				< 0.05^*^			< 0.05^*^
Yes	119(34.3)	56(44.1)	63(28.6)		75(59.5)	44(19.9)	
No	228(65.7)	71(55.9)	157(71.4)		51(40.5)	177(80.1)	
Albumin(g/L)				< 0.05^*^			< 0.05^*^
< 35	112(32.3)	66(52.0)	46(20.9)		64(50.8)	48(21.7)	
≥ 35	235(67.7)	61(48.0)	174(79.1)		62(49.2)	173(78.3)	
EBV				0.165			0.074
Positive	224(64.6)	88(69.3)	136(61.8)		89(70.6)	135(61.1)	
Negative	123(35.4)	39(30.7)	84(38.2)		37(29.4)	86(38.9)	
Extra-nodal Involvement				< 0.05^*^			< 0.05^*^
> 1	163(47.0)	83(65.4)	80(36.4)		82(65.1)	81(36.7)	
0, 1	184(53.0)	44(34.6)	140(63.6)		44(34.9)	140(63.3)	
Elevated LDH level				< 0.05^*^			< 0.05^*^
Yes	222(64.0)	99(78.0)	123(55.9)		100(79.4)	122(55.2)	
No	125(36.0)	28(22.0)	97(44.1)		26(20.6)	99(44.8)	
Elevated β_2_-MG level				< 0.05^*^			< 0.05^*^
Yes	222(64.0)	100(78.7)	122(55.5)		97(77.0)	125(56.6)	
No	125(36.0)	27(21.3)	98(44.5)		29(23.0)	96(43.4)	
LY(× 10^9^/L)	1.06(0.1–7.24)	0.7(0.1–2.5)	1.3(0.1–7.24)	< 0.05^*^	0.9(0.1–6.0)	1.2(0.1–7.24)	0.182
MONO(× 10^9^/L)	0.49(0.04–1.83)	0.64(0.08–1.83)	0.45(0.04–1.34)	< 0.05^*^	0.67(0.04–1.83)	0.42(0.04–1.19)	< 0.05^*^
PLT(× 10^9^/L)	178(2–637)	147(14–557)	195.5(2–637)	< 0.05^*^	110(2–537)	213(44–637)	< 0.05^*^
Attainment of CR				< 0.05^*^			< 0.05^*^
Yes	117(33.7)	18(14.2)	99(45.0)		18(14.3)	99(44.8)	
No	230(66.3)	109(85.8)	121(55.0)		108(85.7)	122(55.2)	

*IPI* International Prognostic Index, *ECOG* Eastern Cooperative Oncology Group, *PTCL* Peripheral T-cell lymphoma, *PTCL-NOS* PTCL-not otherwise specifified, ENKTL extra-nodal NK/T-cell lymphoma, nasal type, *AITL* angioimmunoblastic T-cell lymphoma, *ALCL,ALK* + anaplastic lymphoma kinase positive, *ALCL,ALK − *anaplastic lymphoma kinase negative, *MEITL* monomorphic epitheliotropic intestinal T-cell lymphoma, *SPTCL* subcutaneous panniculitis-like T-cell lymphoma, *HSTCL* hepatosplenic T-cell lymphoma, *MF/SS* mycosisfungoides/Sezary’s syndrome, *EBV* Epstein-barr virus, *LDH* lactic dehydrogenase, *β2-MG* beta-2 micro-globulin, *LY* lymphocyte, *MONO* monocyte, *PLT* platelet, *CR* complete response

^*^Significantly different

Categorical variables are expressed in frequency and percentage (n, %); Continuous variables are expressed in median with range of minimum to maximum

### Cut-off value for LMR/PMR

The optimal cut-off of LMR and PMR were obtained by calculating the area under the curve by receiver operating characteristic (ROC) curve analysis by taking survival as the state variable.

### Statistical analysis

Post hoc power analyses were conducted with GPOWER (Faul, Erdfelder, Lang, & Buchner, 2007) in order to estimate the probability of occurrence of effects in the sample. Through the normal distribution test, the continuous variables included in this study all conform to the normal distribution. The continuous variables such as lymphocyte count, monocyte count and platelet count were shown as median with range and were compared by Mann–Whitney U-test. Other continuous variables were grouped according to the usual clinical threshold and were presented as frequencies and percentages (n, %) in company with categorical variables. All hierarchical and categorical variables were compared by Pearson's chi-square test. Among them, histological subtypes were performed bonferroni-post-hoc-correction. Kaplan–Meier curve was used to analyze OS and log-rank test was used for comparison. Univariate and multivariate logistic regression models were used to evaluate the correlation between clinical variables and complete remission (CR). Cox proportional hazard regression model was used to analyze univariate and multivariate of OS. Statistical analysis was performed by SPSS 23.0 software package. In all comparisons, the results were considered to be statistically significant when the p value was < 0.05, and 95% confidence interval (CI) was given.

## Results

### Patients characteristics

**Fig. 1:**
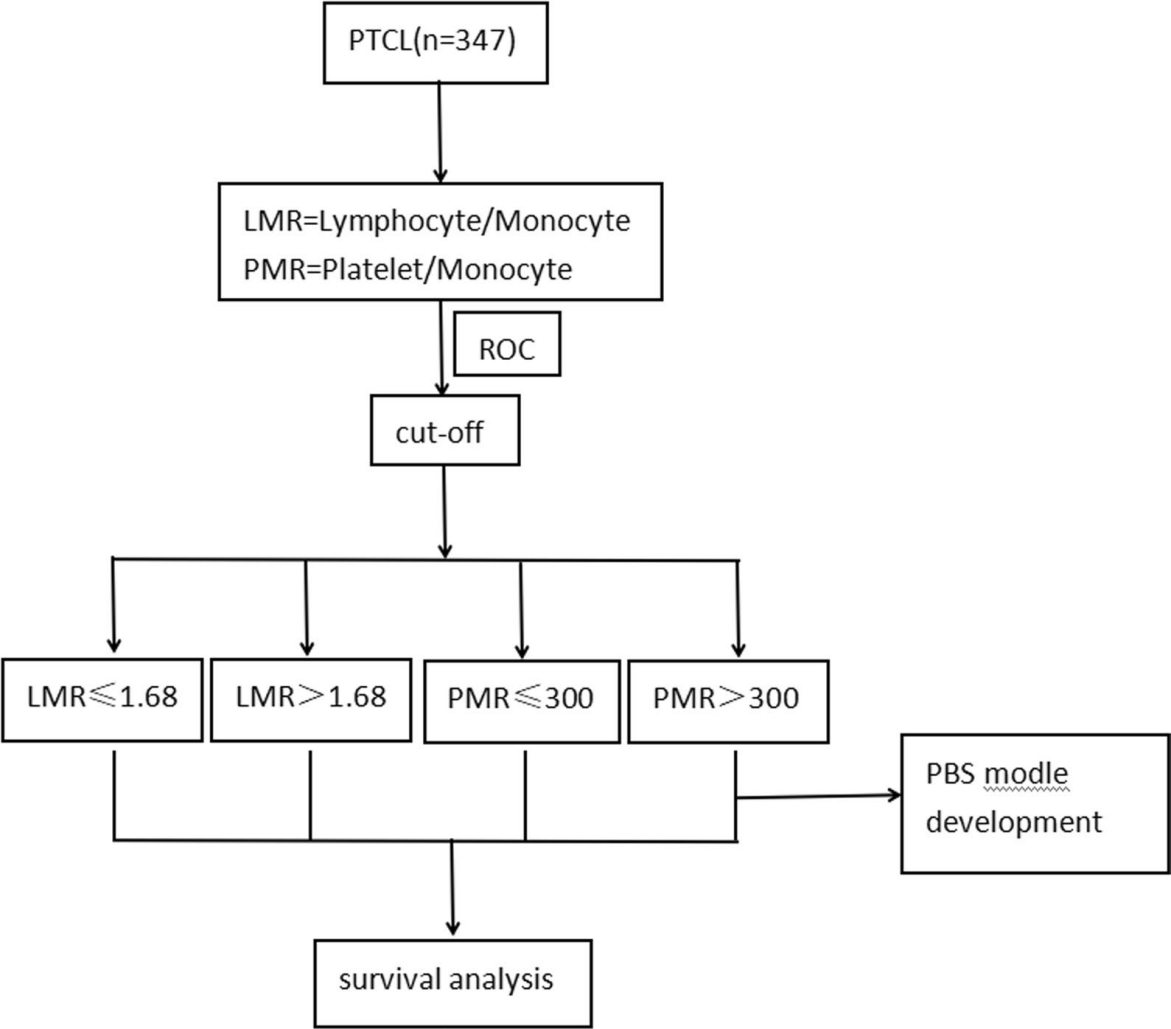
Flow chart of patients included in the analysis

According to the admission conditions, we enrolled 347 patients with newly diagnosed PTCL for analysis and used to establish a prognostic model (Fig. 1). The clinical characteristics and laboratory data were listed in Table 1. The four most common subtypes of PTCL-NOS, ENKTL, AITL and ALCL accounted for 32.0%, 32.5%, 20.2% and 11.8%, respectively. Other rare subtypes including MEITL, SPTCL, HSTCL and MF/SS accounted for 3.5% totally. However, the different subtypes of PTCL did not show statistical significance in the stratification of LMR in our study, either by Pearson's chi square test (P = 0.330) or performing bonferroni-post-hoc-correction (P = 0.570). Although the difference of case typing in PMR was statistically significant through Pearson's chi square test (P = 0.018), the difference was not statistically significant in bonferroni-post-hoc-correction (P = 0.139). The median age at diagnosis was 55 years (rang: 15–84 years), and among them, 36% of the elderly patients were over 60 years old. The ratio of male to female was close to 2:1. About 81.3% of patients were in stage III-IV and 58.5% of them had B symptoms. At the first diagnosis, 32.3% of patients had albumin below 35 g/L, and 64.6% of them were infected with Epstein-Barr virus (EBV). In addition, 163 patients had more than one extra-nodal site involved. The serum lactic dehydrogenase (LDH) and beta-2 micro-globulin (β2-MG) levels were increased in 64% of the patients, respectively.

All patients received at least two cycles of chemotherapy. Except for all ENKTL patients received the protocol containing Pegaspargase. The rest of patients included in this study were treated with CHOP (cyclophosphamide-doxorubicin-vincristine-prednisone) or CHOP-like chemotherapy. Furthermore, 36 patients proceeded to hemopoietic stem cell transplantation, in which 24 of them accepted autologous stem cell transplantation.

### Threshold setting and the relationship between LMR/PMR and clinical parameters and complete remission (CR)

**Fig. 2:**
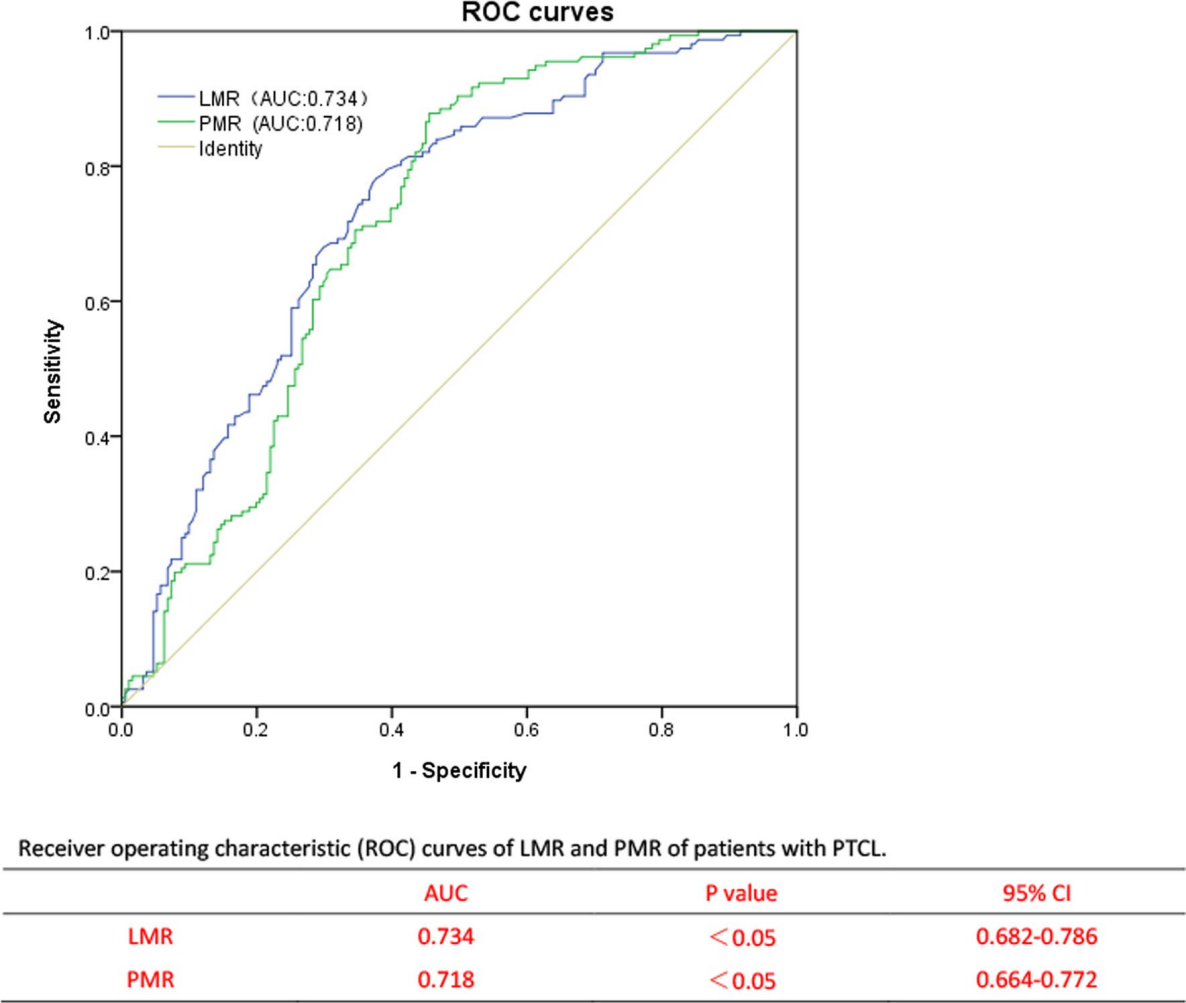
Receiver operating characteristic (ROC) curves of LMR and PMR of patients with PTCL

The ROC curve was generated to select the appropriate cutoff values for LMR and PMR based on the survival analysis (Fig. 2). For LMR, the area under curve (AUC) was 0.734 (95% CI: 0.682–0.786), with a generated maximum joint sensitivity and specificity at the value of 1.68. In addition, for PMR the AUC was calculated to be 0.718 (95% CI: 0.664–0.772), with a generated maximum joint sensitivity and specificity at the value of 300. Table 1 compared the clinical characteristics of patients with LMR and PMR at different levels. The high group and low group were defined as being greater than the cutoff value and less or equal to than the cutoff value, respectively. Post hoc analysis demonstrated sufficient power to distinguish the significant differences (power = 0.996). Patients with LMR ≤ 1.68 or PMR ≤ 300, only 14.2% and 14.3% of them achieved complete response (CR) after treatment. Therefore, it is not difficult to speculate that patients with LMR ≤ 1.68 or PMR ≤ 300 may have poor therapeutic effect.

In univariate logistic regression analysis, lower CR rate was related to first diagnosis older than 60 years, IPI ≥ 3, ECOG ≥ 3, stage III-IV, B symptoms, bone marrow involvement, Albumin < 35 g/L, EBV infection, Extra-nodal > 1, lymphocyte (LY)(× 10^9^/L) < 0.8, monocyte (MONO)(× 10^9^/L) > 1, platelet (PLT)(× 10^9^/L) < 83, elevated LDH and elevated β2-MG, LMR ≤ 1.68 and PMR ≤ 300 (Table 2). Nevertheless, in the multivariate logistic regression analysis, only first diagnosis older than 60 years(OR = 4.031, 95% CI 2.021–8.041, p < 0.001), ECOG ≥ 3(OR = 3.610, 95% CI 1.572–8.290, p = 0.002), stage III-IV(OR = 2.737, 95% CI 1.255–5.969, p = 0.011), bone marrow involvement(OR = 2.581, 95% CI 1.173–5.683, p = 0.018) and EBV infection(OR = 2.090, 95% CI 1.170–3.734, p = 0.013) were statistically significant (Table 2).

**Table 2:** Univariate and multivariate logistic regression models of complete response (CR) in PTCL patients

Covariate	Univariate analysis			Multivariate analysis		
	OR	95%CI	p-value	OR	95%CI	p-value
Sex, Male	1.590	1.001–2.527	0.050			
Age, ≥ 60 years	3.257	1.927–5.505	< 0.05^*^	4.031	2.021–8.041	< 0.05^*^
IPI,3–5	5.818	3.561–9.506	< 0.05^*^			
ECOG,3–5	7.666	3.810–15.423	< 0.05^*^	3.610	1.572–8.290	< 0.05^*^
Stage,III-IV	7.195	3.955–13.088	< 0.05^*^	2.737	1.255–5.969	< 0.05^*^
B symptoms	2.389	1.516–3.766	< 0.05^*^			
Bone marrow	5.613	3.077–10.237	< 0.05^*^	2.581	1.173–5.683	< 0.05^*^
Involvement Albumin, < 35 g/L	2.993	1.742–5.141	< 0.05^*^			
EBV,Positive	2.233	1.409–3.541	0.05	2.090	1.170–3.734	< 0.05^*^
Extra-nodal, > 1	3.118	1.936–5.021	< 0.05^*^			
LY(× 10^9^/L) < 0.8	3.565	2.042–6.224	< 0.05^*^			
MONO(× 10^9^/L) > 1	2.610	0.969–7.028	0.058			
PLT(× 10^9^/L) < 83	7.847	2.759–22.318	< 0.05^*^			
Elevated LDH	2.263	1.429–3.585	< 0.05^*^			
Elevated β2-MG	2.672	1.683–4.243	< 0.05^*^			
LMR ≤ 1.68	4.955	2.816–8.717	< 0.05^*^	1.996	0.906–4.397	0.086
PMR ≤ 300	4.869	2.767–8.567	< 0.05^*^	1.851	0.873–3.924	0.108

*OR* odds ratio, *CI* confidence interval

^*^Significantly different

### The association of LMR/PMR with OS

**Fig. 3:**
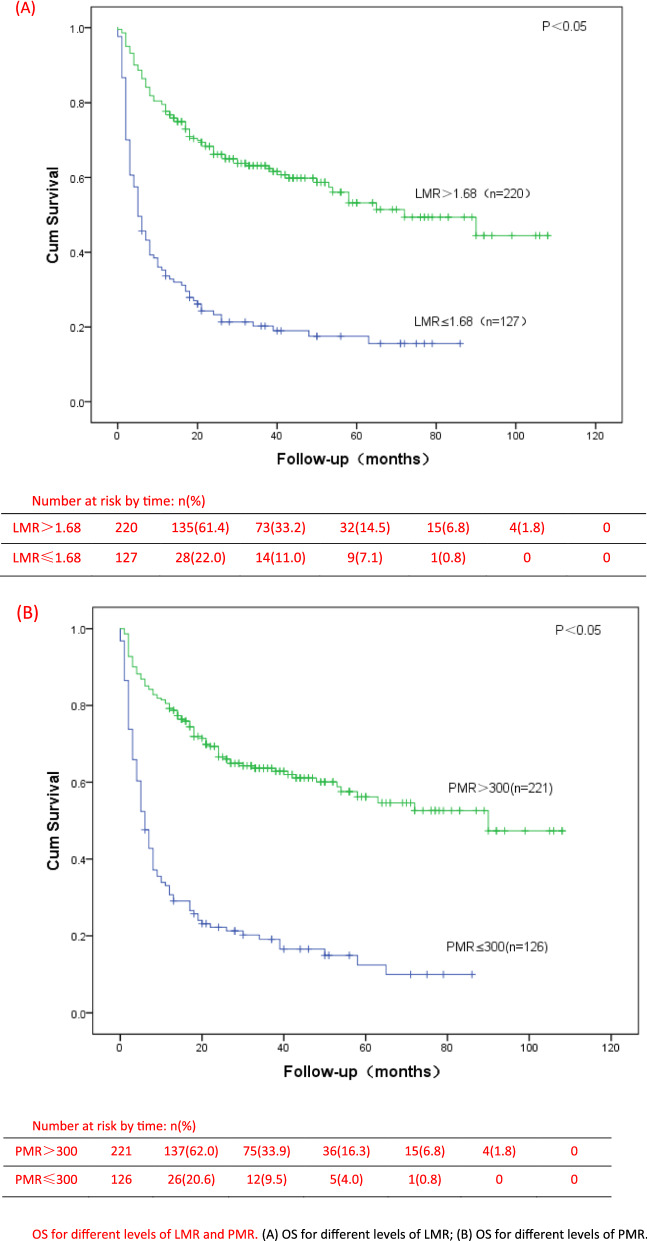
OS for different levels of LMR and PMR. **A** OS for different levels of LMR; **B** OS for different levels of PMR

**Fig. 4:**
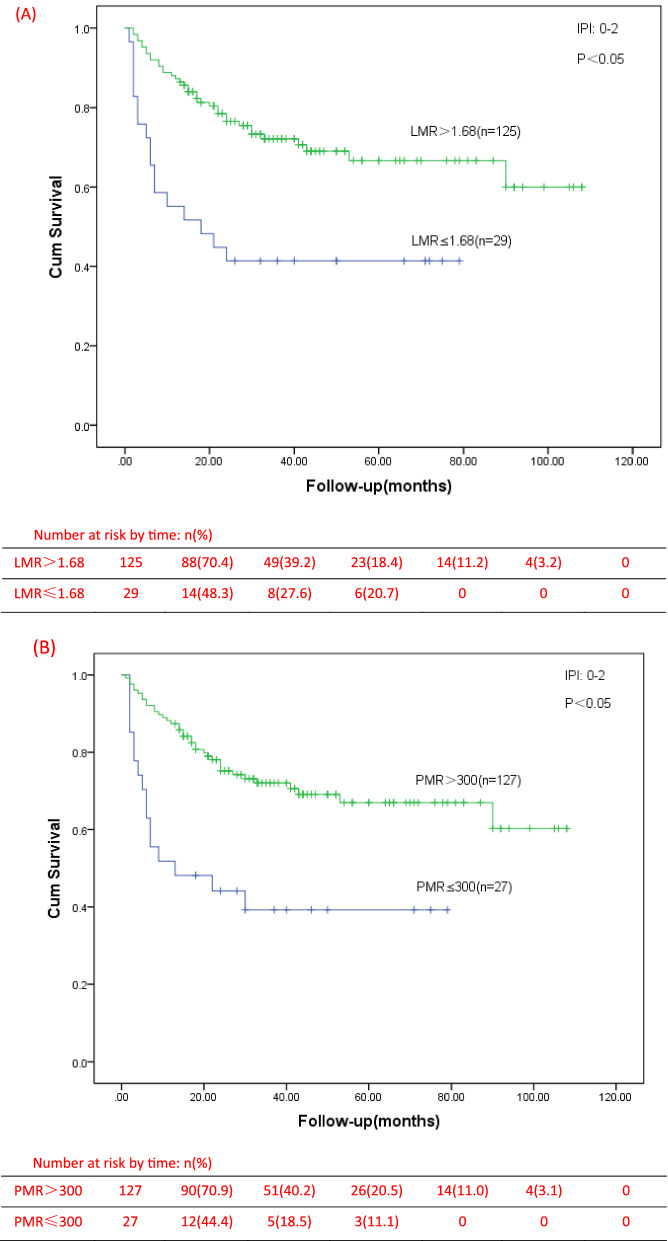

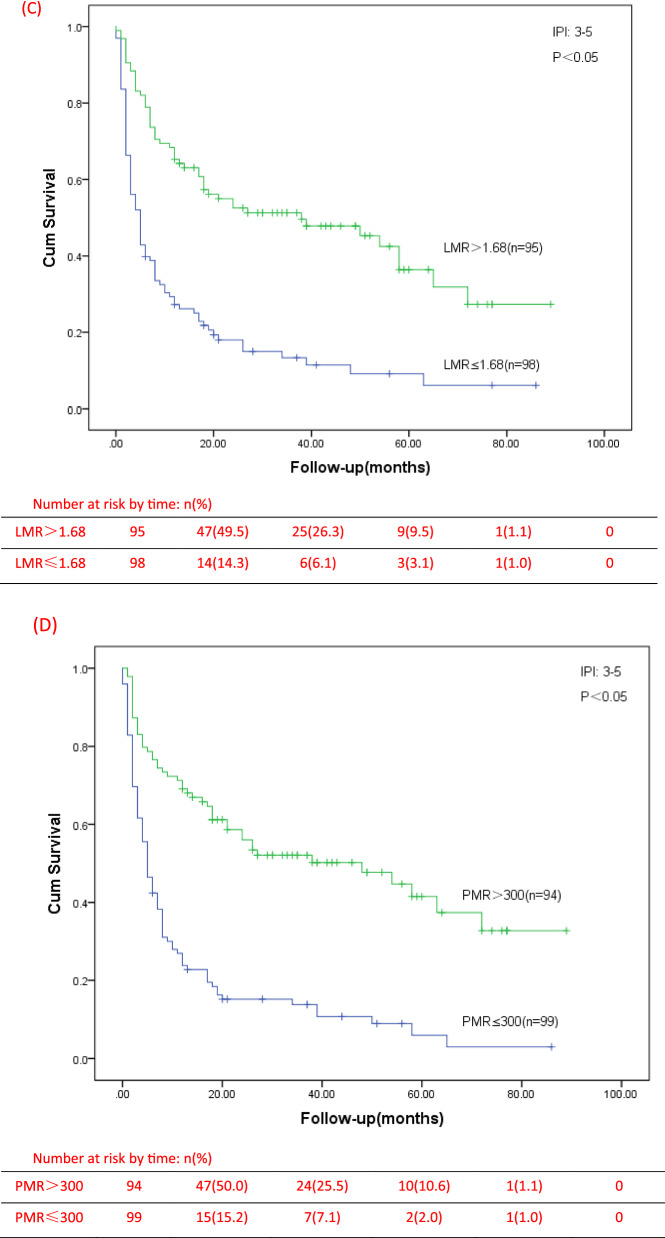
OS for different IPI risk stratification of PTCL patients. **A** OS of patients with different LMR levels in group of IPI score 0–2; **B** OS of patients with different PMR levels in group of IPI score 0–2; **C** OS of patients with different LMR levels in group of IPI score 3–5; **D** OS of patients with different PMR levels in group of IPI score 3–5

In our study, we analyzed the survival of low and high groups of LMR and PMR patients, and found that there were significant differences in OS between all the two groups (P < 0.001) (Fig. 3). The median survival time in the groups with LMR ≤ 1.68 and LMR > 1.68 were 5 months and 28.5 months, and in the groups with PMR ≤ 300 and PMR > 300 were 6 months and 28 months, respectively. The univariate analysis of Cox model showed that first diagnosis older than 60 years, IPI ≥ 3, ECOG ≥ 3, stage III-IV, B symptoms, bone marrow involvement, decreased albumin, EBV infection, Extra-nodal > 1, LY(× 10^9^/L) < 0.8, MONO(× 10^9^/L) > 1, PLT(× 10^9^/L) < 83, elevated LDH, elevated β2-MG, LMR ≤ 1.68 and PMR ≤ 300 were prognostic indicators of OS (Table 3). Then, multivariate analysis was showed only ECOG ≥ 3 (HR = 2.351, 95% CI 1.647–3.356, p < 0.001), stage III-IV (HR = 3.276, 95% CI 1.512–7.099, p = 0.003), Extra-nodal > 1 (HR = 1.659, 95% CI 1.125–2.445, p = 0.039), LMR ≤ 1.68 (HR = 1.751, 95% CI 1.158–2.647, p = 0.006), and PMR ≤ 300 (HR = 1.762, 95% CI 1.201–2.586, p = 0.002) were independent prognostic factors for low OS. Moreover, we found that in the low LMR or PMR groups, the proportion of patients with IPI at 3–5 points is higher. Whether in the low-risk group (IPI = 0–2) or the high-risk group of IPI (IPI = 3–5), the OS of PTCL patients with low LMR group or low PMR group were significantly lower than those of patients in the high LMR group or high PMR group (Fig. 4).

**Table 3:** Univariate and multivariate Cox proportional hazard regression models for overall survival (OS) in PTCL patients

Covariate	Univariate analysis			Multivariate analysis		
	HR	95%CI	p-value	HR	95%CI	p-value
Sex, Male	1.223	0.899–1.662	0.200			
Age, ≥ 60 years	1.379	1.032–1.843	0.030^*^			
IPI, 3–5	3.124	2.265–4.307	< 0.05^*^			
ECOG, 3–5	3.775	2.820–5.054	< 0.05^*^	2.351	1.647–3.356	< 0.05^*^
Stage, III–IV	7.859	3.862–15.993	< 0.05^*^	3.276	1.512–7.099	< 0.05^*^
B symptoms	2.101	1.542–2.862	< 0.05^*^			
Bone marrow	3.062	2.297–4.082	< 0.05^*^			
Involvement Albumin, < 35 g/L	2.209	1.656–2.946	< 0.05^*^			
EBV, Positive	1.390	1.024–1.887	0.035^*^			
Extra-nodal, > 1	3.207	2.374–4.331	< 0.05^*^	1.659	1.125–2.445	0.039^*^
LY(× 10^9^/L) < 0.8	2.279	1.706–3.045	< 0.05^*^			
MONO(× 10^9^/L) > 1	2.292	1.492–3.523	< 0.05^*^			
PLT(× 10^9^/L) < 83	3.459	2.471–4.841	< 0.05^*^			
Elevated LDH	1.613	1.182–2.200	< 0.05^*^			
Elevated β2-MG	2.159	1.560–2.986	< 0.05^*^			
LMR ≤ 1.68	3.496	2.617–4.669	< 0.05^*^	1.751	1.158–2.647	< 0.05^*^
PMR ≤ 300	3.947	2.947–5.287	< 0.05^*^	1.762	1.201–2.586	< 0.05^*^

*HR* hazard ratio, *CI* confidence interval

^*^Significantly different

Considering the heterogeneity of PTCL, we analyzed five common pathological subtypes respectively. Through univariate analysis, it was found that the reduction of LMR and PMR in patients with other common subtypes at the initial diagnosis were significantly correlated with OS except ALCL, ALK + (Tables 4 and 5). This may be related to the relatively small number of this subtype in our study. However, rare types, such as intestinal T cell lymphoma (intestinal lymphoma, commonly seen in B cell lymphoma [17]), incidence rate is low, and the number of cases is very small, so rare types are not analyzed separately.

**Table 4:** The significance of LMR in univariate and multivariate analysis of OS in patients with five major subtypes of PTCL

Histological Subtype-LMR	n	Univariable analysis			Multivariable analysis		
		HR	95%CI	p value	HR	95%CI	p value
PTCL,NOS	111	4.525	2.738–7.475	< 0.05^*^	2.691	1.175–6.162	0.019^*^
ENKTL	113	4.820	2.854–8.138	< 0.05^*^	1.027	0.464–2.276	0.947
AITL	70	1.908	1.013–3.591	0.045^*^	1.785	0.844–3.775	0.129
ALCL,ALK +	20	5.131	0.570–46.172	0.145	–	–	–
ALCL,ALK −	21	7.102	1.740–28.982 ^*^	<0.05^*^	1.415	0.120-16.636	0.782

*HR* hazard ratio, *CI* confidence interval

^*^Significantly different

**Table 5:** The significance of PMR in univariate and multivariate analysis of OS in patients with five major subtypes of PTCL

Histological Subtype-PMR	n	Univariable analysis			Multivariable analysis		
		HR	95% CI	p value	HR	95% CI	p value
PTCL,NOS	111	4.106	2.468–6.830	< 0.05^*^	2.010	0.941–4.293	0.071
ENKTL	113	4.732	2.777–8.064	< 0.05^*^	2.260	1.062–4.812	0.034^*^
AITL	70	3.807	1.986–7.297	< 0.05^*^	2.962	1.313–6.684	< 0.05^*^
ALCL,ALK +	20	8.386	0.927–75.846	0.058	–	–	–
ALCL,ALK −	21	8.046	1.594–40.602	0.012^*^	1.622	0.090–29.096	0.743

*HR* hazard ratio, *CI* confidence interval

^*^Significantly different

### Establishment of PBS model and its correlation with OS

**Fig. 5:**
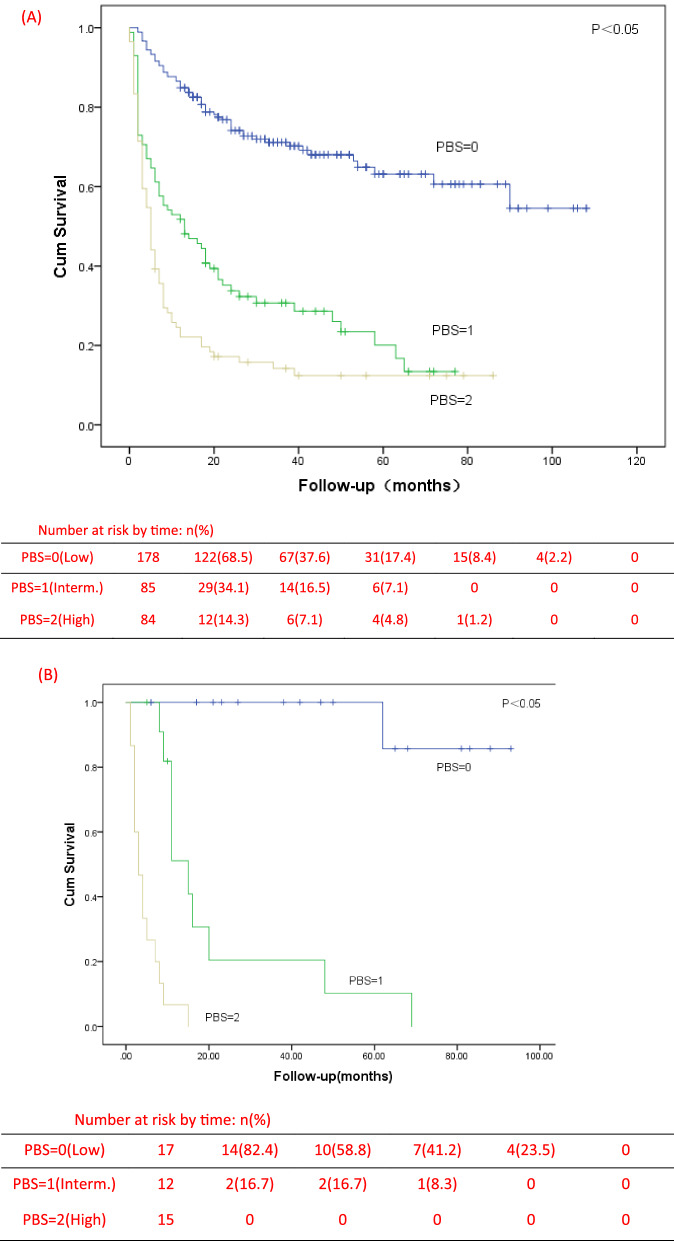
Kaplan–Meier curves of overall survival by risk groups identified by the PBS model in the training sample (n = 347) (**A**) and in the validation sample (n = 44) (**B**). Interm., intermediate

LMR ≤ 1.68 or PMR ≤ 300 were counted as 1 point and LMR > 1.68 or PMR > 300 were counted as 0 point by detecting the peripheral blood cells count at the initial diagnosis. Patients were divided into three groups: PBS 0 group, PBS 1 group and PBS 2 group. 0 was low-risk group, 1 was intermediate risk group, and 2 was high risk group. The OS of these three groups were statistically analyzed by Kaplan–Meier curve and log-rank test. It was found that the OS of low-risk, intermediate risk and high risk patients were significantly different. The median OS of the three groups were 32.5 months, 13 months and 5 months respectively (P < 0.001) (Fig. 5, (A)). Not only that, in the PBS high risk group, 77.4% of the patients survived for less than 1 year, and only 13.1% survived for more than 3 years. In the low-risk group with PBS 0 score, 86% of the patients survived more than 1 year, and 43.4% of the patients survived for more than 3 years, nearly half of them.

### External validation

In order to verify the PBS model, we collected 44 patients who met the inclusion criteria in Huzhou Hospital of Zhejiang University from November 2013 to March 2021. The final follow-up time was August 2021. The median age of onset was 65 years (range: 32–86), including 24 males and 20 females. The median follow-up was 11 months (range: 1–93). During this period, 59.1% of patients died due to disease progression or other causes. After substitution into the PBS model, there were 17 cases in the low-risk (38.6%), 12 cases in the intermediate-risk (27.3%) and 15 cases in the high-risk (34.1%). The 1-year OS of the three groups were 88.2%, 41.7% and 6.7%, respectively. The 3-year OS were 64.7%, 16.7% and 0, respectively. Although there were differences in the shape of Kaplan–Meier curves between the validation sample and the training sample, there were significant differences in OS among the three groups (Fig. 5). The difference of curve shape may be related to the small number of validation samples and the discontinuity of data.

## Discussion

At present, there is no standardized treatment for PTCL in the world. Whether in first-line treatment, second-line treatment or salvage treatment, the prognosis of PTCL is very poor. There is an urgent need to use accurate predictive models to classify patients at risk. Among all the previously reported indices, IPI and PIT are the most commonly used. It is worth noting that there is considerable overlap in the parameters used to establish the various models and that the patients need to be evaluated by imaging, examination, bone marrow, etc.. The operations are complex, and the establishments of these scoring models are not entirely based on PTCL, so their accuracy are questionable.

With the development of medical science in recent years, more and more attention had been paid to the study of tumor molecular mechanisms, especially in tumor micro-environment. Therefore, the research on PTCL has opened a new chapter.

Gene-expression profiling analysis showed that the clinical behavior of tumors could be determined by the characteristics of the tumor cells and interacted with non-neoplastic cells [18]. A growing body of research has consistently shown that tumor-associated inflammatory response is a key determinant of prognosis in cancer patients [19]. ALC is an important indicator of host immune status and was also included in the IPS model used to evaluate the prognosis of patients with Hodgkin's lymphoma [20]. Monocytes can produce a variety of cytokines, such as TNF-α、 IL-1, IL-10 and IL-6 to promote tumorigenesis, angiogenesis and distant metastasis [21]. In previous retrospective studies, we found an closely association between increased serum Interleukin-10 levels and low survival and early recurrence in patients with PTCL [22]. It also indirectly confirmed that lymphocytes and monocytes were closely related to the prognosis of PTCL.

About 2–9% of peripheral leukocytes are peripheral blood monocytes (PBMC), but only 40% of them are used for monocyte circulation, while 60% of monocytes migrate [23]. However, some immature PBMC can differentiate into specialized, tissue-specific macrophages and Antigen-presenting cells (APC). Their differentiation directly determines their functions. The differentiated monocytes/macrophages (Mphi) plays a specific role in the cell mediated innate immunity against infection, immunoregulation, morphogenetic remodelling and malignancy or tissue repair [24, 25]. Zhu et al. [9] found that LMR was associated with TILs/TAMs (Tumor-associated macrophages) ratio, and that low LMR had worse OS in patients with esophageal squamous cell carcinoma. It suggests that a systemic inflammatory response may reflect concurrent focal inflammation in the tumor.

Iacono et al. retrospectively analyzed 165 patients with advanced melanoma. The severity and prognosis of the disease were assessed. The decrease of LMR suggests short OS and more distant metastatic sites in malignant melanoma [10]. Wang et al. reported 355 cases of diffuse large B-cell lymphoma (DLBCL). In the low LMR group, PFS and OS were shorter and M2-TAM content was higher. These results suggested that weak anti-tumor immunity may be an adverse prognostic factor for aggressive lymphoma, identified in high-risk patients [14]. Thus, lymphocyte counts, monocyte counts, and LMR surrogate markers of tumor micro-environment had been reported as prognostic factors for B cell lymphoma [14–16]. Similarly, recent studies had shown that both lymphocyte counts and monocyte counts could predict the clinical outcome of T-cell lymphomas [24–26]. And patients with T-cell lymphomas after autologous peripheral hematopoietic stem cell transplantation had longer OS and PFS with autograft lymphocyte-to-monocyte ratio (A-LMR) greater than or equal to 1. Compared with patients with A-LMR less than 1, the five years OS rate was 87% to 26%, and the five years PFS rate was 72% to 16%, significant difference [26]. Feng et al. [27] Studied 75 newly diagnosed T-lymphoblastic lymphoma (T-LBL) patients and found that patients with LMR ≤ 2.8 had both inferior progression-free survival (PFS) and inferior overall survival (OS), in which the differences were much more remarkable in the international prognostic index score 0–2 subgroup. However, the cutoff value of LMR in our study is inconsistent with the result by Feng et al. Feng et al. mainly focused on the T-LBL patients, while our work included the five most common subtypes and four rare subtypes of PTCL. Perhaps, the different inclusion criteria led to the different cutoff values of LMR. For all that, LMR is still an independent prognostic factor for affecting the survival of PTCL patients. Suman Ghosh et al. Present the case of a T-LBL with superior vena cava syndrome, developing tumor lysis syndrome on instituting definitive chemotherapy in a young patient. From the patient's blood cell count, although we cannot know the accurate monocyte values, it is not difficult to speculate that the patient's LMR is greater than 1.68 and the prognosis is relatively good, which is also consistent with the good follow-up clinical outcome of the patient mentioned in the article [28].

The average platelet count in humans is between 150 × 10^9^ and 400 × 10^9^ per liter, but over time, the number of individual platelets remains the same [29]. Platelets mainly participates in the organism haemostasis and the thrombosis. In recent years, there are increasing evidences that platelets and tumor cells have significant cross-communication, suggesting that they play an important role in the progression of malignant tumors, the occurrence of tumor-associated local inflammation, and cancer-associated thrombosis. On the one hand, tumors can affect the RNA profile of platelets, the number of circulating platelets and their activation status. On the other hand, tumor-induced platelets contain a large number of active biomolecules, including platelet-specific and circularly ingested biomolecules that are released upon activation of platelets and are involved in the development of malignant tumors [30].

Platelet activation plays an important role in tumor-associated immune thrombosis and multiple metastasis. Activated platelets were known to secrete a range of inflammatory chemokines that activate inflammatory signaling pathways in white blood cells, including PAF, RANTES, CCL3, CXCL1, CXCL4 (platelet factor 4), and CXCL7 [31, 32]. Serotonin (5-hydroxytryptamine) is another platelet-releasing product that may affect monocyte function. Monocytes exposed to 5-hydroxytryptamine showed increased NF-κB activation, increased cytokine production induced by LPS, and decreased apoptosis, possibly due to changes in BCL-2 or MCL-1 expression [33]. Białas et al. [13] Found that the ratio of monocyte to platelet can be used as a prediction tool for pulmonary embolism in acute deteriorate of chronic obstructive pulmonary disease (AECOPD). In the past few decades, a large number of clinical studies have shown that daily aspirin can reduce the incidence, metastasis and mortality of tumors, especially for colorectal cancer [34]. In addition, platelets are closely associated with the complement system [35]. To date, platelets have been suggested as an adverse predictor of survival in PTCL Mechanistically, thrombocytopenia impairs the immune response, increases the risk of bleeding, and reflects the bone marrow failure, indicting a poor prognosis in patients with PTCL [36]. Recently, Guillem-Llobat and his collaborators demonstrated in an immunodeficiency mouse model that low-dose aspirin reduces the metastasis of lung cancer by avoiding the enhanced pro-aggregation effect caused by platelet-tumor cell interaction [37]. These clinical studies have fully confirmed that platelets are closely related to the occurrence and development of PTCL.

In recent years, many clinical studies had shown that hematological components of the systemic inflammatory response, including the lymphocyte-to-monocyte ratio (LMR), the platelet-to-moncyte ratio (PMR) and the systemic immune inflammation index (SII) are efficient prognostic indicators in patients with cancers. The decrease of LMR has been confirmed by many clinical studies to be related to the poor prognosis of esophageal cancer, melanoma, Hodgkin's lymphoma, multiple myeloma and DLBCL [9, 10, 38–40]. PMR has also been proved to play an important role in the early warning of pulmonary embolism in patients with AECOPD [13]. But most of the studies did not investigate the reference intervals (RIs) of these parameters in healthy controls. Luo et al. Conducted a retrospective cohort study of 5969 Chinese healthy people aged 18 to 79 by retrieving the data from the health routine examination center database and laboratory information system of four participating centers in Western China. They found that the individual's gender can significantly influence LMR. Surprisingly, they also found that with an increase in age, the LMR tend to decrease. The RIs of LMR was 2.63–9.9 [41]. Another healthy population study from Iran. They included the data of 2212 healthy subjects and the average age was 47.9 ± 9.29 years. The mean value of LMR was 11.15 ± 3.14 [42]. There are significant differences between the two values above for healthy people, which may be related to the age and population differences of subjects. However, regardless of the reference range, the cut-off value of LMR obtained in our study is significantly lower than the reference ranges. Unfortunately, the RIs of PMR in normal population has not been clarified.

## Conclusions

The present study showed the correlation between readily available peripheral blood biomarkers and survival in patients with PTCL. LMR ≤ 1.68 and PMR ≤ 300 were significantly associated with lower CR rate and poorer OS and were regarded as independent prognostic factors by univariable and multivariable analysis. The PBS model based on LMR and PMR can well distinguish high-risk patients, and we have also been well verified after substituting the clinical data of another center. Compared with the current IPI and PIT models, our PBS model is more simple, inexpensive and widely available, which could help improve the risk stratification and guide clinicians to make better treatment strategies.

Our study still has some limitations. Firstly, the retrospective study may be biased in the selection of patients. Secondly, the dynamic changes of patients during treatment did not taken into account during analysis. Third, our sample size is relatively small and lack of cytogenetic data. Another issue is the cut-off value of LMR/PMR used in clinical practice. In the past and in our study, the ROC curve based on survival was used to determine the optimal cut-off value, indicating that there was inconsistency between the centers. Therefore, further exploration and prospective trials with larger samples are needed in the future and the PBS model we established also needs further verification.

## Data Availability

All data included in our manuscript were real clinical data. However, because this was a retrospective clinical study, the original blood samples have been unable to obtain, so the data used were all obtained from the previous patients' in-hospital tests, without repeated tests.

## Abbreviations

PTCL: Peripheral T-cell lymphoma
NHL: Non-Hodgkin’s lymphoma
CR: Complete response
OS: Overall survival
ECOG: Eastern cooperative oncology group
LMR: Lymphocyte-monocyte ratio
PMR: Platelet-monocyte ratio
PBS: Peripheral blood score
CBC: Blood cell count
PTCL-NOS: Peripheral T-cell lymphoma, not otherwise specified
ENKTL: Extra-nodal natural killer (NK)/T cell lymphoma, nasal type
AITL: Angioimmunoblastic T-cell lymphoma
ALK + ALCL: Anaplastic lymphoma kinase positive anaplastic large cell lymphoma
ALK − ALCL: Anaplastic lymphoma kinase negative anaplastic large cell lymphoma
MEITL: Monomorphic epitheliotropic intestinal T-cell lymphoma
SPTCL: Subcutaneous panniculitis like T-cell lymphoma
MF/SS: Mycosisfungoides/Sezary’s syndrome
HSTCL: Hepatosplenic T-cell lymphoma
IPI: International Prognostic Index
LDH: Lactic dehydrogenase
TAMs: Tumor-associated macrophages
ROC: Receiver operating characteristic
CI: Confidence interval
EBV: Epstein-Barr virus
β2-MG: Beta-2 microglobulin
LY: Lymphocyte
MONO: Monocyte
PLT: Platelet
PBMC: Peripheral blood monocytes
APC: Antigen-presenting cells
Mphi: Monocytes/macrophages
DLBCL: Diffuse large B-cell lymphoma
PFS: Progression free survival
A-LMR: Autograft lymphocyte-to-monocyte ratio
HSC: Hematopoietic stem cells
TF: Tissue factor
